# Polymorphisms in *Plasmodium falciparum* dihydropteroate synthetase and dihydrofolate reductase genes in Nigerian children with uncomplicated malaria using high-resolution melting technique

**DOI:** 10.1038/s41598-020-80017-6

**Published:** 2021-01-12

**Authors:** Adeyemi T. Kayode, Fehintola V. Ajogbasile, Kazeem Akano, Jessica N. Uwanibe, Paul E. Oluniyi, Philomena J. Eromon, Onikepe A. Folarin, Akintunde Sowunmi, Dyann F. Wirth, Christian T. Happi

**Affiliations:** 1 African Centre of Excellence for Genomics of Infectious Diseases, Redeemer’s University, Ede, Nigeria; 2 Department of Biological Sciences, Redeemer’s University, Ede, Nigeria; 3 Institute of Advanced Medical Research and Training, College of Medicine, University of Ibadan, Ibadan, Nigeria; 4 Department of Pharmacology and Therapeutics, University of Ibadan, Ibadan, Nigeria; 5 Department of Immunology and Infectious Diseases, Harvard T.H. Chan School of Public Health, Boston, MA USA

**Keywords:** Parasite genomics, Antimicrobial resistance, Malaria

## Abstract

In 2005, the Nigerian Federal Ministry of Health revised the treatment policy for uncomplicated malaria with the introduction of artemisinin-based combination therapies (ACTs). This policy change discouraged the use of Sulphadoxine-pyrimethamine (SP) as the second-line treatment of uncomplicated falciparum malaria. However, SP is used as an intermittent preventive treatment of malaria in pregnancy (IPTp) and seasonal malaria chemoprevention (SMC) in children aged 3–59 months. There have been increasing reports of SP resistance especially in the non-pregnant population in Nigeria, thus, the need to continually monitor the efficacy of SP as IPTp and SMC by estimating polymorphisms in dihydropteroate synthetase (*dhps*) and dihydrofolate reductase (*dhfr*) genes associated with SP resistance. The high resolution-melting (HRM) assay was used to investigate polymorphisms in codons 51, 59, 108 and 164 of the *dhfr* gene and codons 437, 540, 581 and 613 of the *dhps* gene. DNA was extracted from 271 dried bloodspot filter paper samples obtained from children (< 5 years old) with uncomplicated malaria. The *dhfr* triple mutant I_51_R_59_N_108_, *dhps* double mutant G_437_G_581_ and quadruple *dhfr* I_51_R_59_N_108_ + *dhps* G_437_ mutant haplotypes were observed in 80.8%, 13.7% and 52.8% parasites, respectively. Although the quintuple *dhfr* I_51_R_59_N_108_ + *dhps* G_437_E_540_ and sextuple *dhfr* I_51_R_59_N_108_ + *dhps* G_437_E_540_G_581_ mutant haplotypes linked with *in-vivo* and *in-vitro* SP resistance were not detected, constant surveillance of these haplotypes should be done in the country to detect any change in prevalence.

## Introduction

In 2005, the Nigerian Federal Ministry of Health (FMoH) revised the treatment policy for uncomplicated malaria with the introduction of artemisinin-based combination therapies (ACTs)^1^. This treatment policy change discouraged the use of Chloroquine (CQ) and Sulphadoxine-pyrimethamine (SP) as the first-line and second-line treatment of uncomplicated falciparum malaria, respectively. However, SP continues to be used as an intermittent preventive treatment of malaria in pregnancy (IPTp) and seasonal malaria chemoprevention (SMC) in children aged 3–59 months in malaria-endemic countries including Nigeria^2,3^.

Providing evidence for the continued use of SP as IPTp and SMC in the context of SP resistance in Africa requires constant epidemiological surveillance of parasite resistance levels by monitoring polymorphisms in genes associated with SP resistance. Point mutations such as S_436_A, A_437_G, K_540_E, A_581_G and A_613_T/S in dihydropteroate synthetase (*dhps*) gene and N_51_I, C_59_R, S_108_N and I_164_L in dihydrofolate reductase (*dhfr*) gene are observed to play significant roles in SP resistance^4–6^. At a population level, the quintuple mutation in *P. falciparum* parasites, i.e., triple *dhfr* mutations of I_51_, R_59_, and N_108_, plus double *dhps* mutations of G_437_, and E_540_ (I_51_R_59_N_108_G_437_E_540_) has also been strongly linked with reduced SP efficacy as IPTp, reduced parasite clearance ability in asymptomatic pregnant women and shortened post-treatment prophylactic activity^7–9^.

Since the deployment of SP in Nigeria as IPTp in 2001^10^ and SMC in 2013^11^, there have been various reports of these point mutations in both *dhfr* and *dhps* genes associated with SP resistance in various parts of the country^11,12^. Reports of high prevalence of the triple mutant genotype of *dhfr* (N_51_I, C_59_R and S_108_N) in addition to the A_437_G mutation in the *dhps* gene is common in Nigeria^13^. However, occurrence of the quintuple *dhfr* + *dhps* mutation comprising of N_51_I, C_59_R and S_108_N + A_437_G and K_540_E is scarce^13^.

The goal of this study was, therefore, to: (i) examine the current status of circulating *Dhfr* and *Dhps* haplotypes by describing polymorphisms in codons 51, 59, 108 and 164 of *Dhfr* and codons 437, 540, 581 and 613 of *Dhps* and (ii) estimate the prevalence of single, double, triple, quadruple and quintuple mutation^4^ in parasites present in the five (5) Nigerian States.

## Results

### Baseline demographics and clinical data

A total of 271 children from Adamawa (50), Bayelsa (45), Imo (82), Kwara (45) and Sokoto (49) states with uncomplicated falciparum malaria were considered in this analysis. Baseline characteristics of the children are shown in Table 1. Overall, 160 (59.04%) were male. Mean age of all children included in the study was 38.2 ± 16.5 months. Also, mean enrollment body temperature was 37.5 ± 2.5 °C, and 17 of the 271 children (4.4%) had hyperpyrexia (enrollment temperature > 40 °C). Overall geometric asexual parasitemia was 14,673parasite/μL^−1^ (range: 2003–198,200).

**Table 1:** Demographic and clinical features of children with uncomplicated *Plasmodium falciparum* infection.

	Adamawa (n = 50)	Bayelsa (n = 45)	Imo (n = 82)	Kwara (n = 45)	Sokoto (n = 49)	All states (n = 271)
**Gender**						
Male/Female	28/22	29/16	43/39	30/15	30/19	160/111
**Age (month)**						
Mean (SD)	35.1(15.7)	35.3(15.8)	46.8(14.4)	36 (15.7)	31.4(16.8)	38.2(16.5)
**Weight (Kg)**						
Mean (SD)	12.1(3.5)	13.8(3.9)	16.2(2.8)	12.4(4.2)	9.9(3.2)	13.3(4.1)
**Temperature (°C)**						
Mean (SD)	38.3(0.6)	37.5(1.2)	36.9(4.2)	37.8(1.3)	37.6(1.3)	37.5(2.5)
**Haematocrit (%)**						
Mean (SD)	31(4.5)	29.5(5.4)	30.3(3.8)	32.3(6.5)	28.3(5.8)	30.3(5.2)
No. with anaemia	22	18	32	11	21	104
Mild	22	14	31	10	15	92
Moderate	0	4	1	1	6	12
Severe	0	0	0	0	0	0
**Parasitaemia (µL**^**−1**^**)**						
Geometric mean	16,493	10,304	20,359	33,072	4827	14,673
Range	2047–195,000	2052–109,040	2008–195,947	2071–198,200	2003–28,860	2003–198,200
No. ≥ 100,000	5	2	10	13	0	30

### Merozoite surface protein genotyping of *Plasmodium falciparum*

The family-specific polymorphic length markers of *msp*-2 and *msp*-1 were used for genotyping the *P. falciparum* in children considered for this study. In general, 180 (66.8%) children had polyclonal infections (Table 2). The mean complexity of infection (mCOI) for parasites across all population was 2.3. Allelic family distributions for both *msp*-2 and *msp*-1 per State is represented in Table 2. Overall, the 3D7 allelic family was the most amplified (50.7%) in the *msp-*2 polymorphic marker while the K1 allelic family was the most detected (18.4%) *msp-*1 polymorphic marker.

**Table 2:** Parasite clonality, Complexity of Infection and Allelic family distribution.

State	n	Clonality			Allelic family Distribution^a^									
					*msp*-2 (n = 213)			*msp*-1 (n = 223)						
		Poly	Mono	COI (range)	3D7	FC27	BOTH	K1	MAD20	RO33	K1 + RO33	K1 + MAD20	MAD20 + RO33	ALL
Adamawa	50	37	13	2.8 (1–4)	9	9	24	7	4	5	5	8	5	9
Bayelsa	45	38	7	2.5 (1–4)	18	6	19	4	2	1	2	21	2	8
Imo	82	40	42	1.7 (1–4)	46	10	5	16	20	18	1	2	3	0
Kwara	45	29	16	2.1 (1–5)	18	5	7	10	8	2	9	5	0	1
Sokoto	49	36	13	2.8 (1–5)	17	6	14	4	2	8	18	0	5	8
All	271	180	91	2.3 (1–5)	108	36	69	41	36	34	35	36	15	26

^a^All 271 samples were characterised using the polymorphic markers. Some samples were amplified using both polymorphic markers while some were amplified either by msp-2 or msp-1.

### Polymorphisms in the *dhfr* gene

**Figure 1:**
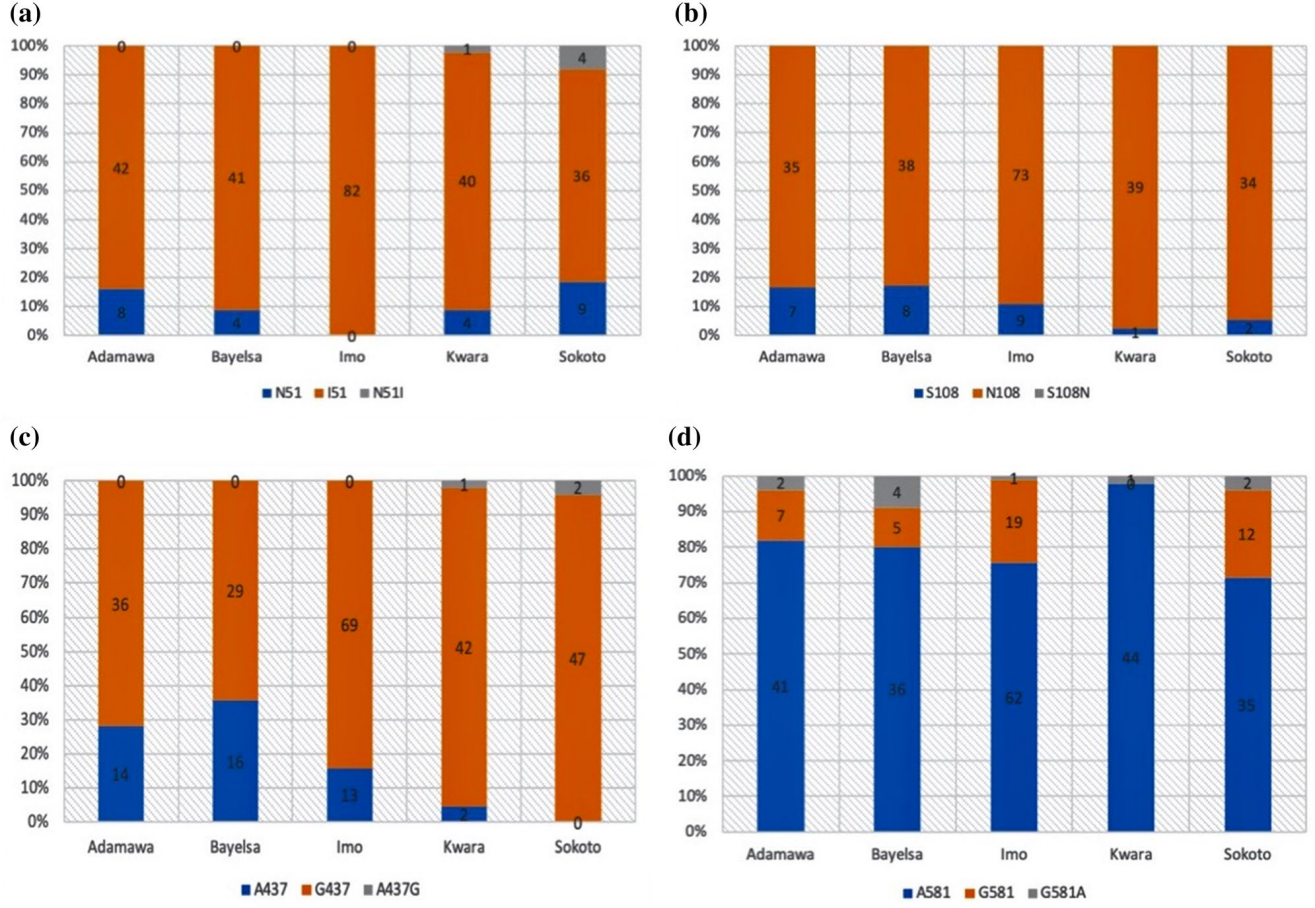
Bar charts showing frequencies of wild, mutant and mixed allelic infections in *dhfr* (**a**) Codon 51 (**b**) Codon 108 and *dhps* (**c**) Codon 437 and (**d**) Codon 581.

From the 271 children considered, 241 (88.9%), 245 (90.4%) and 219 (80.8%) were infected with parasites that harboured the mutant I_51_, R_59_ and N_108_ alleles respectively. None of the children was infected with parasites habouring the mutant L_164_ allele. Distribution of the mutant I_51_, R_59_ and N_108_ alleles were significantly higher in polyclonal infections than in monoclonal infections (p < 0.05 for each allele). All isolates obtained from children enrolled in Imo State were infected with parasites that harboured only the mutant I_51_ and R_59_ alleles (Fig. 1a,b).

### Polymorphisms in the *dhps* gene

From the 271 children considered, 223 (82.3%) and 45 (16.6%) were infected with parasites that harboured the mutant G_437_ and G_581_ alleles respectively. None of the children was infected with parasites that harboured the mutant E_540_ and T/S _613_ alleles_._ Distribution of the mutant G_437_ and G_581_ alleles were significantly higher in polyclonal infections than in monoclonal infections (p < 0.05 for each allele) (Fig. 1c,d).

### *Dhfr* and *Dhps* haplotype frequencies and distribution

**Figure 2:**
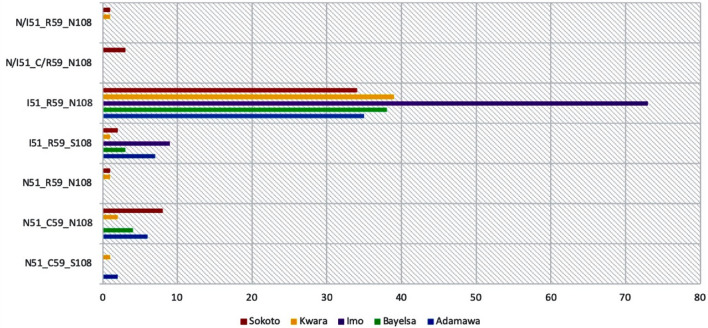
Bar chart showing the distribution of *dhfr* haplotypes in five Nigerian States. Red colour represents Sokoto State, Yellow represents Kwara State, Purple represents Imo State, Green represents Bayelsa State and Blue represents Adamawa State. The triple mutant haplotype I_51_R_59_N_108_ was the most distributed in all five States.

Parasites harbouring the *dhfr* triple mutant I_51_R_59_N_108_, double mutant I_51_R_59_, double mutant R_59_N_108,_ and single mutant N_108_ haplotypes were observed in 80.8%, 8.1%, 0.74% and 7.4% respectively of the 271 children considered (Fig. 2). The proportions of children with *dhfr* triple mutant I_51_R_59_N_108_ and double mutant I_51_R_59_ haplotypes in Northern (Adamawa, Sokoto and Kwara) and Southern (Bayelsa and Imo) States was similar (p > 0.05). Conversely, the proportion of children with *dhfr* single mutant N_108_ was significantly higher in Northern States versus the Southern States (p > 0.05). Double mutant R_59_N_108_ haplotype was only recorded in two children from Kwara and Sokoto States (Northern States) (Fig. 2).

**Figure 3:**
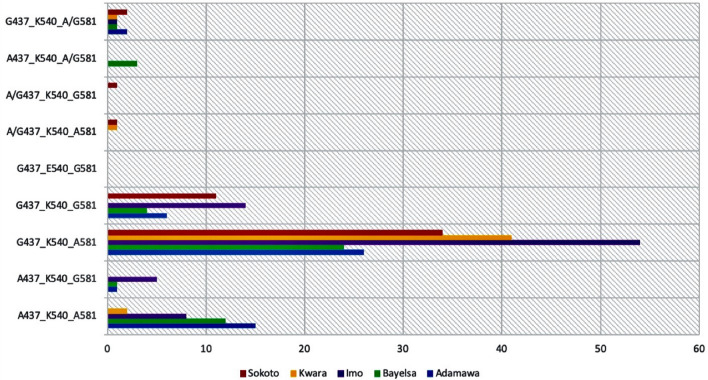
Bar chart showing the distribution of *dhps* haplotypes in five Nigerian States. Red colour represents Sokoto State, Yellow represents Kwara State, Purple represents Imo State, Green represents Bayelsa State and Blue represents Adamawa State. The single mutant haplotype G_437_K_540_A_581_ was the most distributed in all five States.

Parasites harbouring the *dhps* double mutant G_437_G_581_, single mutant G_437_ and single mutant G_581_ haplotypes were observed in 13.7%, 66.1% and 2.6% respectively of the 271 children considered (Fig. 3). There was no triple and quadruple *dhps* mutant haplotype because none of the isolates harboured mutant allele of *dhps* 540. There was no difference in the distribution of the double mutant G_437_G_581_ and single mutant G_437_ haplotypes in the Northern and Southern States (p > 0.05).

### Prevalence of combined *dhps* and *dhfr* haplotypes

The prevalence of different alleles on *dhfr* and *dhps* genes when combined as single mutant haplotype (*dhfr* N_108_), double mutant haplotype (*dhfr* R_59_N_108_), triple mutant haplotype (*dhfr* I_51_R_59_N_108_), quadruple mutant haplotype (*dhfr* I_51_R_59_N_108_ + *dhps* G_437_), quintuple mutant haplotype (*dhfr* I_51_R_59_N_108_ + *dhps* G_437_E_540_) and sextuple mutant haplotype (*dhfr* I_51_R_59_N_108_ + *dhps* G_437_E_540_G_581_) were considered (Table 3). Although most of the *dhfr* I_51_R_59_N_108_ triple mutant haplotype was observed in polyclonal infections as only eight were observed in monoclonal infections, there was no significant difference in distribution based on clonality (p > 0.05) or location, i.e., Northern and Southern States (p > 0.05). The quadruple mutant haplotype was observed in 143 (52.8%) of the 271 children (Table 3). Eighty-three (58.0%) of the 143 were observed in polyclonal infections but there was no significant difference in the distribution of this haplotype based on clonality (polyclonal vs. monoclonal) (p > 0.05) or location, i.e., Northern and Southern States (p > 0.05). None of the 271 children was infected with parasites harbouring the quintuple mutation and sextuple mutation as the E_540_ mutant allele was absent (Table 3).

**Table 3:** Prevalence of combine *dhfr* + *dhps* mutant haplotypes.

Mutation	Haplotype	Adamawa (n-50)	Bayelsa (n = 45)	Imo (n = 82)	Kwara (n = 45)	Sokoto (n = 49)	Total (n = 271)
Single (%)	*dhfr* N_108_	3 (6.0)	0	0	0	0	3 (1.1)
Double (%)	*dhfr* R_59_N_108_	0	0	0	1 (2.2)	1 (2.0)	2 (0.7)
Triple (%)	*dhfr* I_51_R_59_N_108_	7 (14.0)	11 (24.4)	8 (9.7)	2 (4.4)	0	28 (10.3)
Quadruple (%)	*dhfr* I_51_R_59_N_108_ + *dhps* G_437_	19 (38.0)	19 (42.2)	47 (57.3)	35 (77.8)	23 (46.9)	143 (52.8)

## Discussion

This study assessed the status of circulating *dhfr* and *dhps* haplotypes by describing polymorphisms on codons 51, 59, 108 and 164 of *dhfr* gene and codons 437, 540, 581 and 613 of *dhps* gene and estimated the prevalence of *dhfr* + *dhps* combined mutant haplotypes in 271 parasites obtained from children (< 5 years) children with uncomplicated falciparum malaria in Nigeria 10 years after treatment policy was changed to ACTs.

Polymorphism data from our study showed high prevalence of mutant I_51_ (88.9%) and N_108_ (80.8%) *dhfr* alleles and mutant G_437_ (82.3%) *dhps* allele. Similar prevalence of these mutant *dhfr* and *dhps* alleles have been recorded in Nigeria^11,13^ and other West African countries^14,15^. Although prevalence of these mutant alleles are generally high in West Africa^16^, lower prevalence (26.5–56.25) have been recorded in other West African countries^4,17^. The exact reason for the difference in prevalence amongst these West African countries may be as a result of the varied use of SP in these countries^18^. Also, *P. falciparum* and other disease etiologies exist as co-infections in patients in these areas. It is equally plausible that the use of other sulpha-related drugs in the treatment of these co-infections may select for these mutations in the *P. falciparum* genome at varying levels^19^.

Sulfadoxine-pyrimethamine was previously used as weekly prophylaxis for malaria during which the mutant E_540_ allele was recorded^2,5^. However, our study which was conducted when the therapeutic use of SP had changed from weekly prophylaxis to IPTp and SMCs, showed the absence of the mutant E_540_ allele. Similar trend has been observed in recent studies in Nigeria^11,19^. The supposed disappearance of this mutant allele is perhaps, as a result of the reduced SP drug pressure in the country due to this treatment policy change.

Pearce et al.^20^ stated the importance of measuring the frequency of haplotypes as against the prevalence of each point mutation separately as haplotypes are determinants of resistance levels. We observed a high frequency (80.8%) of the *dhfr* triple mutant haplotype (I_51_R_59_N_108_) which suggests the persistent circulation of similar parasites as those reported in earlier studies post-ACT introduction^11,12,18^. These parasites are probably selected for as a result of the SP drug pressure, as this drug was not completely withdrawn in the country but rather used as IPTp and SMCs till date. The use of drugs such trimethoprin sulfamethazole targeting *dhfr* genes in *Pneumocystis carnii* in an environment where malaria and HIV coinfections is common, could also be responsible for the selection of this haplotype in *Plasmodium falciparum* populations in Nigeria^5^. The occurrence of this mutant haplotype at such a high frequency is worrisome as such triple mutations in the *dhfr* gene has been associated with a 1.5- to threefold higher pyrimethamine resistance in vitro than I_51_N_108_ or R_59_N_108_ double mutations^5^. Thus, the efficacy of pyrimethamine as a partner drug in SP’s use as IPTp and SMCs is threatened. Although the double *dhps* mutant haplotype (G_437_G_581_) was observed in our study (12.9%), the absence of the E_540_ mutation in the *dhps* gene in combination with either the single mutant G_437_ or double mutant G_437_G_581_ in this study is desired as the double mutant G_437_E_540_ haplotype is essential for sulfadoxine resistance^5^.

We also observed high levels of polyclonal infections in this study as most of the States considered had > 60% proportion of children with polyclonal infections. Further analysis of data revealed that the mutant *dhfr* (I_51_, R_59_ and N_108_) and mutant *dhps* (G_437_ and G_581_) alleles were significantly higher in polyclonal infections than in monoclonal infections (p < 0.05 for each mutant allele). Also, the *dhfr* triple mutant I_51_R_59_N_108_ haplotype was significantly higher in polyclonal infections (p < 0.05). These observations may be problematic as high levels of polyclonality is linked to increased parasite transmission and diversity. This may result in the increase in spread of these mutant alleles and haplotype within the country. This can jeopardise the use of SPs as IPTp and SMCs in Nigeria.

Unpublished *P. falciparum* microsatellite data^21^ confirmed the high intra-population diversity observed using the *msp-*1 and *msp*-2 polymorphic genes but also revealed low population differentiation in these parasites from the five parasite populations (Nigeria States). This suggests that, despite the high parasite diversity observed, parasites were genetically similar across the country. This may be responsible for the observed similarities in the distribution of the combined *dhfr* + *dhps* triple mutant haplotype (*dhfr* I_51_R_59_N_108_: p > 0.05) and quadruple mutant haplotype (*dhfr* I_51_R_59_N_108_ + *dhps* G_437_: p > 0.05) in both Northern and Southern States of the country considered in this study.

The quintuple mutant haplotype (*dhfr* I_51_R_59_N_108_ + *dhps* G_437_E_540_) that was earlier reported (2005) in Nigeria^5^ was absent in this study. This is possibly due to the reduced SP drug pressure as a result of policy change from SP as second-line treatment of malaria to ACTs. Both the quintuple and sextuple mutant haplotypes have been strongly linked with in vivo and in vitro SP resistance^22^ in Southern and East Africa^23,24^ and their absence in this current study, is not only beneficial, but consistent with reports from other West African countries^4,25^. Nevertheless, continuous monitoring for re-emergence of *dhfr* I_51_R_59_N_108_ + *dhps* G_437_E_540_ and emergence of *dhfr* I_51_R_59_N_108_ + *dhps* G_437_E_540_G_581_ haplotypes should be maintained in the country to detect any change in the recorded prevalence. This would ensure that alternative control measures are rapidly put in place to prevent the spread of these haplotypes within the country, which if not checked, will lead to reduced efficacy of SP as IPTp and SMCs.

## Methods

### Study site

**Figure 4:**
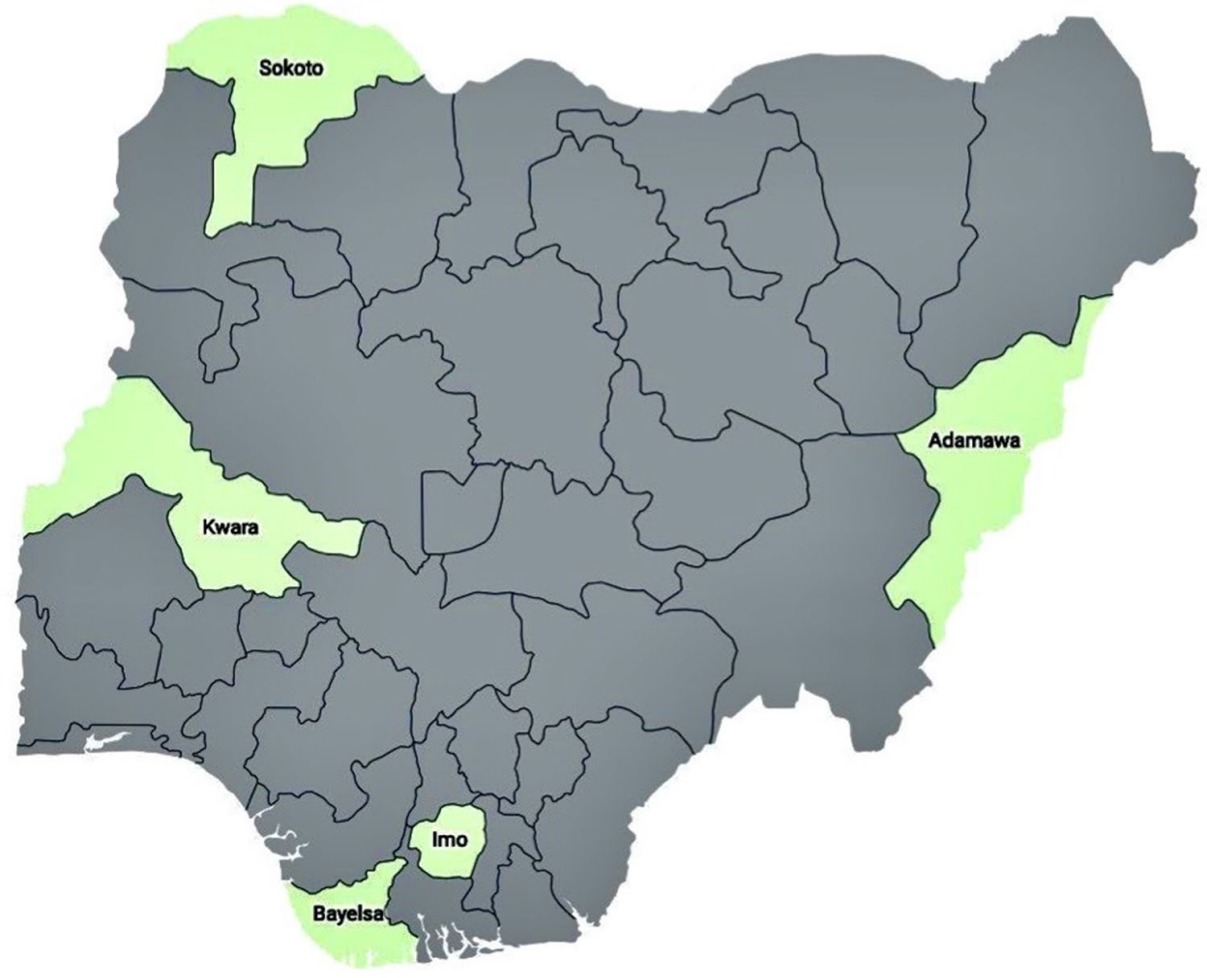
This map shows the States in Nigeria were analysed samples were obtained. These five States represents five of the six geopolitical zones in the country, i.e., North-West: Sokoto, North-East: Adamawa, North-Central: Kwara, South-South: Bayelsa and South-East: Imo. (Map generated using Datawrapper: https://www.datawrapper.de).

This study is part of a national drug therapeutic efficacy testing (DTET) study for monitoring antimalarial efficacies of artemether-lumefantrine (AL), artesunate-amodiaquine (AA) and dihydroartemisinin-piperaquine (DHP) in the treatment of acute uncomplicated falciparum infections in children under the age of five years. Samples considered for analysis in this study were obtained from five Nigerian States which were sub-classified into Northern region (Adamawa, Sokoto and Kwara States) and Southern region (Bayelsa and Imo States) (Fig. 4). These States are parts of sentinel locations for the National Malaria Elimination Program (NMEP) of the Federal Ministry of Health in Nigeria for the year 2014/2015 Drug Therapeutic Efficacy Testing (DTET) study^26^.

### Patients enrolment criteria and sample collection

Description of patient enrolment at sentinel locations was initially discussed in an earlier study^26^. Two to three drops of finger-prick blood were blotted on 3 mm Whatman filter paper (Whatman International Limited, Maidstone, United Kingdom) before treatment (Day 0) and during follow-up on days 1–3, 7, 14, 21, 28, 35 and 42 post-treatment. Blood samples impregnated on filter papers were allowed to air-dry appropriately at room temperature and stored in airtight envelopes with silica gels.

### DNA extraction

DNA was extracted from dry blood spot (DBS), i.e., blood impregnated filter papers of day 0 samples (before treatment) for the detection of polymorphisms of the drug resistance markers and to determine parasite genetic diversity as previously described^27^. A Qiagen DNA extraction kit (Qiagen, Hilden, Germany) was used to extract DNA from DBS, following the manufacturer's protocol. Briefly, a quarter of the DBS was used for extraction, and DNA content was eluted in a final volume of 60 μl with buffer AE.

### Genotyping *Plasmodium falciparum* using the *msp*-1 and *msp*-2 gene

The polymorphic length markers *msp*-2 and *msp*-1 were amplified by nested PCR as previously described^28^. The Glurp polymorphic marker was not considered in this study due to the low PCR amplification reported in Nigeria^29^. PCR amplification was performed on a thermocycler (Eppendorf Vapo. Protect Mastercycler pro, Germany) in a final volume of 25 μl. Two per cent (2%) agarose gel was used for the resolution of PCR amplicons. The amplified products were sized against a 100-base pair (bp) DNA molecular weight marker (New England Biolabs, Beverly, MA) and visualised using a gel visualisation box (Syngene, UK). Interpretations were made based on the number of parasite clones present in a sample. Briefly, infections were defined as polyclonal if parasites from a single patient showed more than one allelic family or more than one amplicon fragment in a single allelic family of the gene. Infections were defined as monoclonal if an isolate had a single amplicon fragment in one allelic family and the other allelic family(ies) was (were) not amplified^6^.

### High resolution melting drug resistance assay

High resolution melting (HRM) assay was performed as previously described^30^. Briefly, the 10X primer–probe mix and reaction mix was prepared. One microliter of the quantified DNA sample was dispensed in PCR well containing 9.0 μl reaction mix. PCR cycling and melting conditions used were those described earlier^30^. Standard software included with the instruments was used for unlabeled probe analysis to visualise melting peaks based on different melting temperatures, indicative of different base pairs, and compared with wild type and mutant controls to call alleles for both *dhfr* (Supplementary Figs. S1 and S2) and *dhps* (Supplementary Figs. S3 and S4) assays. Parasite genomic controls used in this study, i.e., 3D7, HB3, DD2 and Tm90C6B were graciously donated by BEI resources (MR4, BEI resources, USA).

### Statistical analysis

Data were double-entered and analysed using version 6 of Epi-Info software and the statistical program SPSS for Windows version 20.0. Proportions were compared by calculating χ^2^ using Yates’ correction, Fisher’s exact or Mantel Haenszel tests. Normally distributed, continuous data were analysed by Student’s t-test and analysis of variance (ANOVA). Mann–Whitney *U* tests and the Kruskal Wallis tests (or by Wilcoxon ranked sum test) were used to compare data that did not conform to normal distribution. P values of < 0.05 were taken to indicate significant differences.

## Ethical approval

The study was conducted in accordance with the Declaration of Helsinki, and the protocol was approved by the National Health Research Ethics Committee, Federal Ministry of Health (FMOH), Abuja, Nigeria. Informed consent was obtained from parents and legal guardians of participants prior to enrollment in study.

## Supplementary Information

Supplementary Figures.
