# Supplementary material for: Polymorphisms in Plasmodium falciparum dihydropteroate synthetase and dihydrofolate reductase genes in Nigerian children with uncomplicated malaria using high-resolution melting technique
Source: Sci Rep. 2021 Jan 12;11:471. doi: 10.1038/s41598-020-80017-6 (PMC7803958; doi:10.1038/s41598-020-80017-6)
Supplement: Supplementary file 1 — Supplementary Figures. [file 41598_2020_80017_MOESM1_ESM.docx]

# **Polymorphisms in *Plasmodium falciparum* Dihydropteroate synthetase and Dihydrofolate reductase genes in Nigerian children with uncomplicated malaria using high-resolution melting technique**

# Adeyemi T. Kayode^1,2^, Fehintola V. Ajogbasile^1,2^, Kazeem Akano^1,2^, Jessica N. Uwanibe^1,2^, Paul E. Oluniyi^1,2^, Philomena J. Eromon^1^, Onikepe A. Folarin^1,^ Akintunde Sowunmi^3,4^, Dyann F. Wirth^5^, and Christian T. Happi*^1, 2,5^

^1^African Centre of Excellence for Genomics of Infectious Diseases, Redeemer's University, Ede, Nigeria

^2^Department of Biological Sciences, Redeemer's University, Ede, Nigeria

^3^Institute of Advanced Medical Research and Training, College of Medicine, University of Ibadan, Nigeria

^4^Department of Pharmacology and Therapeutics, University of Ibadan, Ibadan, Nigeria

^5^Department of Immunology and Infectious Diseases, Harvard T.H. Chan School of Public Health, Boston, MA, USA

*Corresponding Author

Christian T. Happi, PhD

African Centre of Excellence for Genomics of Infectious Diseases,

Department of Biological Sciences, Redeemer's University, Ede, Nigeria

[happic@run.edu.ng](mailto:happic@run.edu.ng)

Supplementary Fig. S1


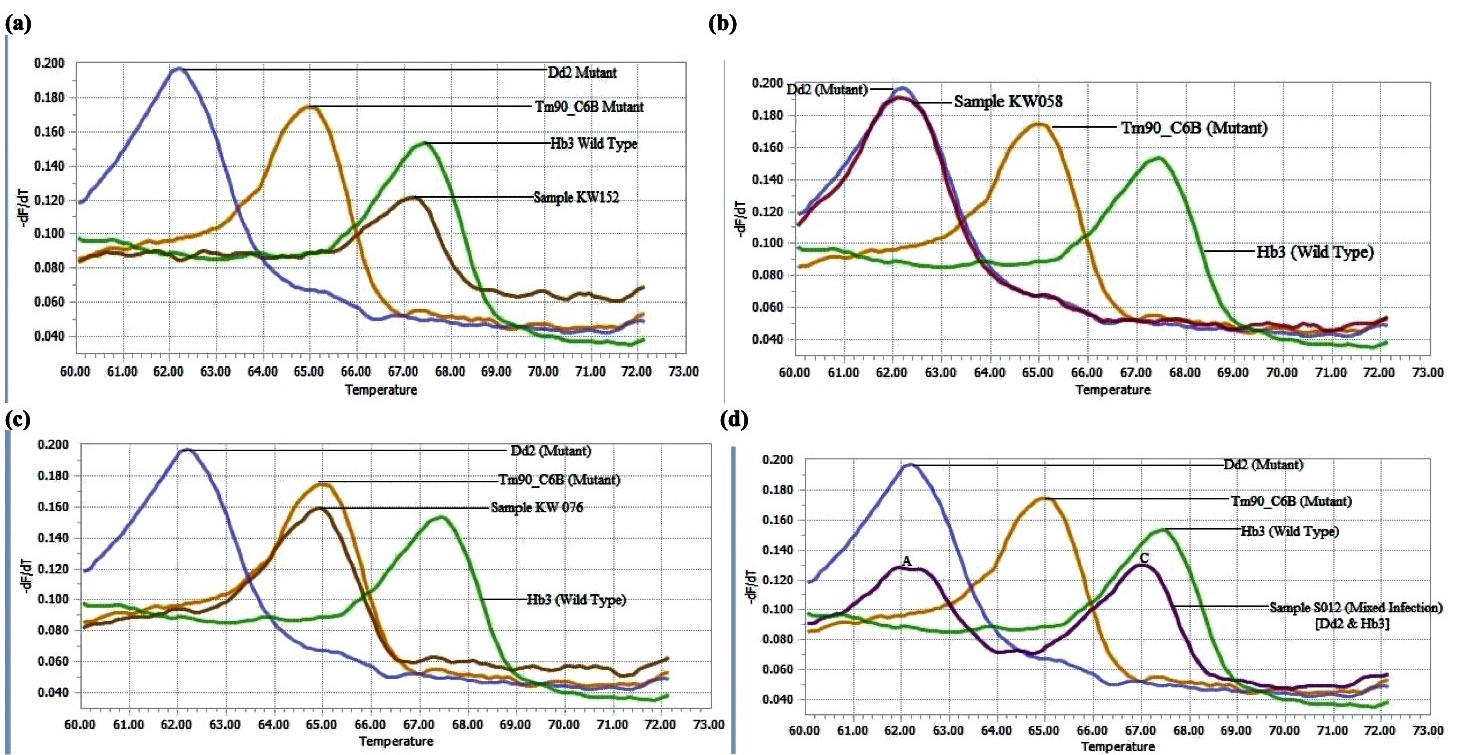


High resolution melt curves of *Dhfr* codon 51.59. In Fig. S1a, sample had identical melt curve as the HB3 wildtype control thus habouring the N_51_ and C_59_ wild type alleles. In Fig. S1b, sample had identical melt curve as the Dd2 mutant control thus habouring the I_51_ and R_59_ mutant alleles. In Fig. S1c, sample had identical melt curve as the TM906CB mutant control thus habouring the N_51_ and R_59_ alleles. In Fig. S1d, sample had identical melt curves as the Dd2 mutant and HB3 wild type controls thus designated as mixed allelic infection.

Supplementary Fig. S2


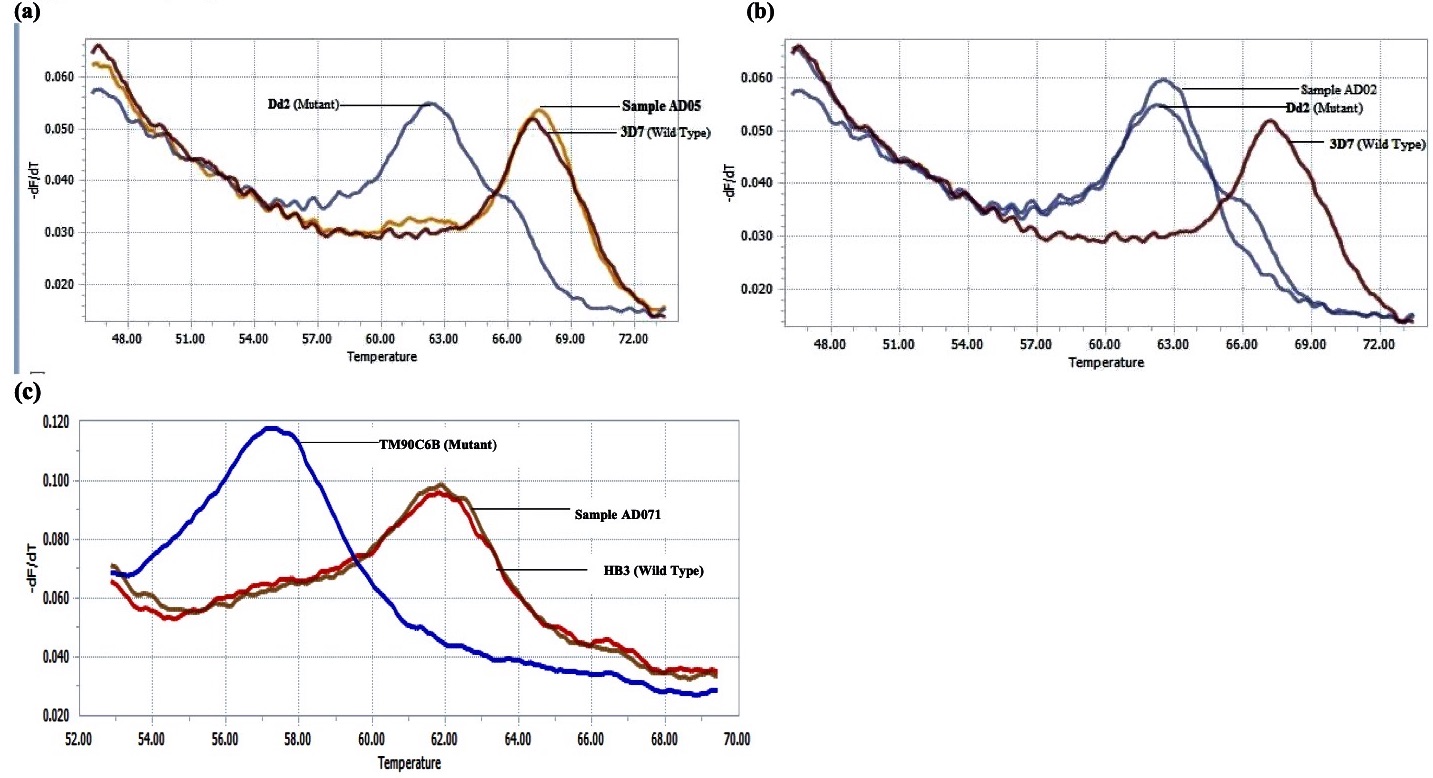


High resolution melt curves of *Dhfr* codon 108 and 164. In Fig. S2a, sample had identical melt curve as the 3D7 wildtype control thus habouring the S_108_ wild type allele. In Fig. S2b, sample had identical melt curve as the Dd2 mutant control thus habouring the N_108_ mutant allele. In Fig. S2c, sample had identical melt curves as the HB3 wild type control thus habouring the I_164_ wild type allele. None of the sample had a melt curve curve as the TM90C6B mutant control.

Supplementary Fig. S3


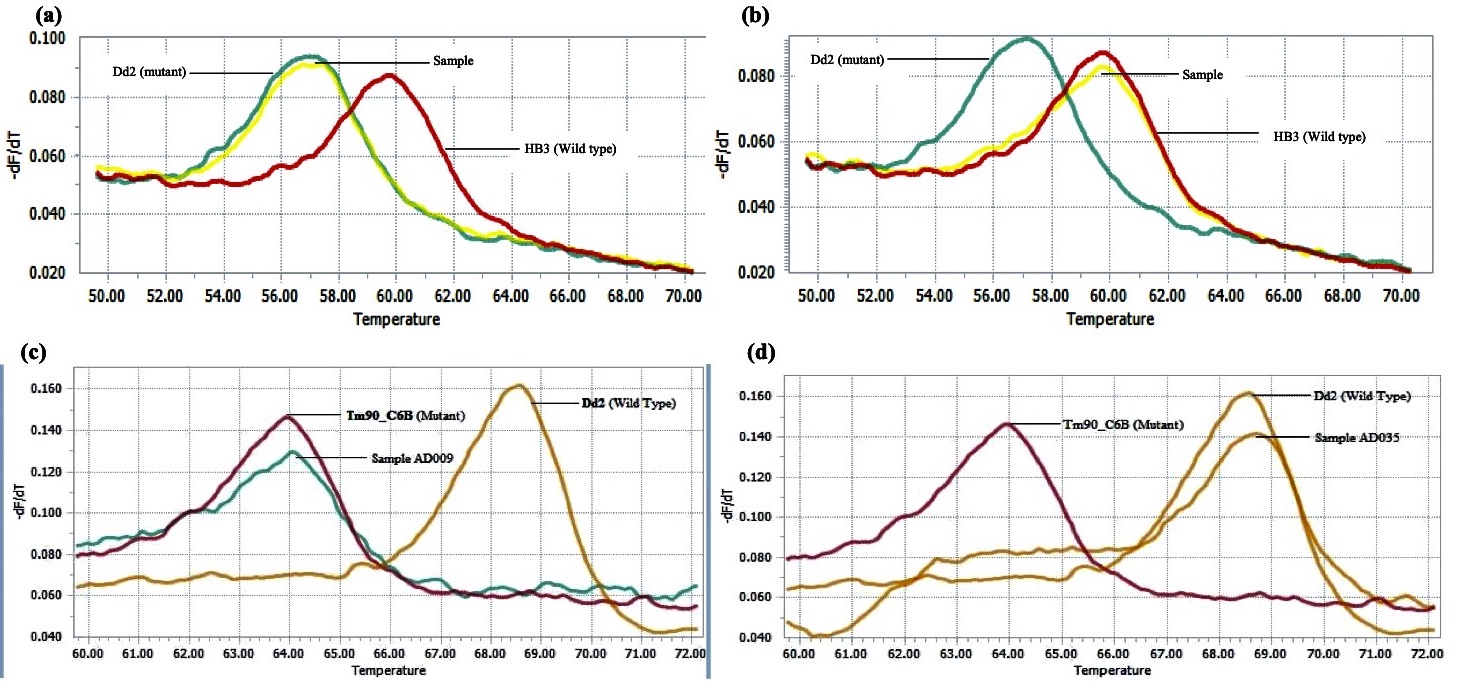


High resolution melt curves of *Dhps* codon 437 and 581. In Fig. S3a, sample had identical melt curve as the Dd2 mutant control thus habouring the G_437_ mutant allele. In Fig. S3b, sample had identical melt curve as the HB3 wild type control thus habouring the S_437_ wild type allele. In Fig. S3c, sample had identical melt curve as the TM906CB mutant control thus habouring the G_581_ mutant allele. In Fig. S3d, sample had a identical melt curves as the Dd2 wild type control thus habouring the A_581_ wild type allele.

Supplementary Fig. S4


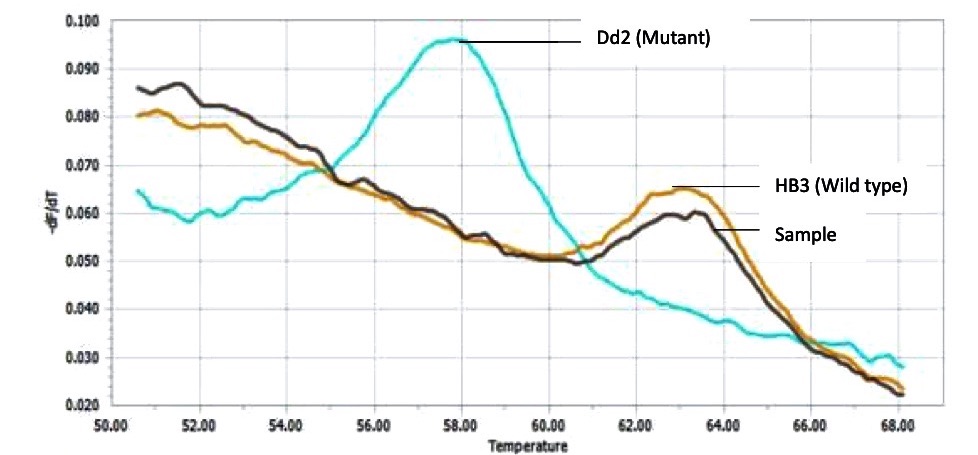


High resolution melt curves of *Dhps* codon 613. Sample had identical melt curve as the HB3 wildtype control thus habouring the A_613_ wild type allele. None of the samples had melt curves identical to the Dd2 mutant control.
